# Plant Functional Diversity and Species Diversity in the Mongolian Steppe

**DOI:** 10.1371/journal.pone.0077565

**Published:** 2013-10-08

**Authors:** Guofang Liu, Xiufang Xie, Duo Ye, Xuehua Ye, Indree Tuvshintogtokh, Bayart Mandakh, Zhenying Huang, Ming Dong

**Affiliations:** 1 State Key Laboratory of Vegetation and Environmental Change, Institute of Botany, Chinese Academy of Sciences, Beijing, China; 2 College of Life and Environmental Sciences, Hangzhou Normal University, Hangzhou, China; 3 Institute of Botany, Mongolian Academy of Sciences, Ulaanbaatar, Mongolia; 4 University of Chinese Academy of Sciences, Beijing, China; University of Saskatchewan, Canada

## Abstract

**Background:**

The Mongolian steppe is one of the most important grasslands in the world but suffers from aridization and damage from anthropogenic activities. Understanding structure and function of this community is important for the ecological conservation, but has seldom been investigated.

**Methodology/Principal Findings:**

In this study, a total of 324 quadrats located on the three main types of Mongolian steppes were surveyed. Early-season perennial forbs (37% of total importance value), late-season annual forbs (33%) and late-season perennial forbs (44%) were dominant in meadow, typical and desert steppes, respectively. Species richness, diversity and plant functional type (PFT) richness decreased from the meadow, via typical to desert steppes, but evenness increased; PFT diversity in the desert and meadow steppes was higher than that in typical steppe. However, above-ground net primary productivity (ANPP) was far lower in desert steppe than in the other two steppes. In addition, the slope of the relationship between species richness and PFT richness increased from the meadow, via typical to desert steppes. Similarly, with an increase in species diversity, PFT diversity increased more quickly in both the desert and typical steppes than that in meadow steppe. Random resampling suggested that this coordination was partly due to a sampling effect of diversity.

**Conclusions/Significance:**

These results indicate that desert steppe should be strictly protected because of its limited functional redundancy, which its ecological functioning is sensitive to species loss. In contrast, despite high potential forage production shared by the meadow and typical steppes, management of these two types of steppes should be different: meadow steppe should be preserved due to its higher conservation value characterized by more species redundancy and higher spatial heterogeneity, while typical steppe could be utilized moderately because its dominant grass genus *Stipa* is resistant to herbivory and drought.

## Introduction

As one of the most important contemporary environmental problems, the loss of biodiversity has become a major topic of concern [1]. On-going climate changes also threaten species diversity in natural ecosystems [2-6], especially in drylands [7]. These changes have caused drastic fragmentation of the landscape, thereby affecting ecosystem properties and services [7-9]. As functional properties of ecosystems are determined by species diversity and vegetation structure, the change of vegetation structure caused by species loss would have negative effects on the functioning of ecosystems [10,11]. Therefore, an enhanced understanding of structure and function of natural vegetation is necessary for maintenance of function in endangered natural ecosystems [12].

In Mongolia, grasslands cover approximately 80% of the land area and comprise a major part of East Asian grasslands [13]. There are three main types of steppes including the meadow, typical and desert steppes, which are all ecologically fragile as they are sensitive to climate changes, anthropogenic disturbances or both [14]. In recent years, overgrazing and cultivation activities have reduced their vegetation cover, which is critical to protect steppes from wind erosion [15]. Unfortunately, these landuses have led to a conversion of vegetation type from typical to desert steppes [16]. These changes have adversely affected the entire (regional) steppe ecosystem [17]. Meanwhile, with the predictable future rise of atmospheric temperature and the likely decline in annual precipitation up to the end of this century [18], the impact of climate change, anthropogenic disturbance and their interaction on vegetation structure and function would be particularly serious in the Mongolian steppe.

To our knowledge, little is known about the composition and diversity of plant communities in this temperate steppe zone [15,19] despite its importance for policy-making or steppe management. Furthermore, rising evidence has shown that species diversity may increase the functional diversity of plant communities [20-23], and the functional diversity can strongly determine the functioning of the ecosystem [24,25] via interspecific facilitation [26] or niche complementarity [27]. Therefore, the understanding of the composition and diversity of plant communities as well as their variation across large spatial scales can strengthen our ability to predict how steppe ecosystems respond to temporal variation in landuses, climate changes or both [28,29].

The objectives of this study were to: 1) investigate the plant species diversity and the functional diversity in the different types of steppes, 2) suggest conservation strategies for these steppes. We surveyed the three types of Mongolian steppe (meadow, typical and desert steppes), and then calculated species diversity and functional diversity of the plant community. Moreover, considering a possible sampling effect on diversity, we hypothesized that the positive correlation between species diversity and functional diversity is independent of steppe types. We predicted the largest slope of this coordination would appear in desert steppe but the lowest would occur in the meadow steppe due to contrasting species-pool sizes in plant community assemblage.

## Materials and Methods

### Study area and sampling design

**Figure 1:**
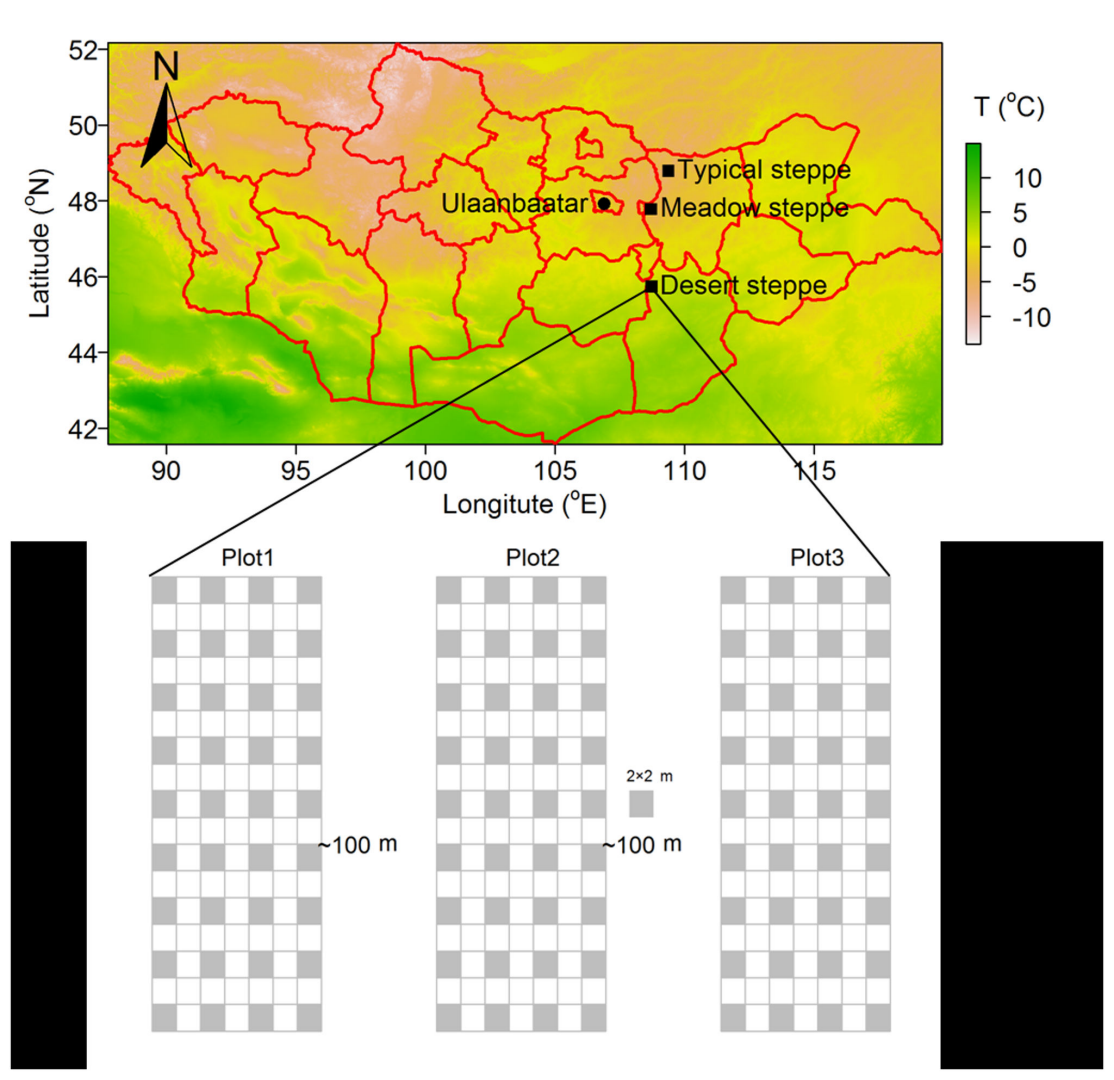
The geographic positions and the layout of plots in three sites belonging to the meadow, typical and desert steppes on the map of Mongolia, respectively. In each site, three 34 m × 14 m plots were interspaced by ca. 100 m and contained 108 subplots of 2 m × 2 m. The filled and open squares in plots stand for subplots and inter-subplot spaces, respectively.

The study was performed in three types of steppes (the desert, typical, and meadow steppes) in the central Mongolian steppe belt (Figure 1), which was authorized by Institute of Botany, Mongolian Academy of Sciences (MAS). They are representative grassland vegetation types and have a wide distribution in Mongolia. Mean annual temperature, mean annual precipitation, soil nitrogen (N), soil phosphorus (P), and soil types were collected (Table 1). The overall % vegetation cover of the desert, typical and meadow steppes were 5-20%, 35-60% and 60-90%, respectively.

**Table 1:** Climate and soil factors (mean ± SE) of three sites belonging to the meadow, typical and desert steppes in Mongolia, respectively.

Environment		Meadow steppe	Typical steppe	Desert steppe
**Climate**				
	MAT (°C)	-2.8	2.0	4.5
	MAP (mm yr^-1^)	273.6	276.4	110.6
**Soil**				
	N (mg kg^-1^) (0-10)	11.48 ± 0.54	6.23 ± 0.22	5.83 ± 0.28
	(10-20)	9.92 ± 0.59	6.04 ± 0.21	5.75 ± 0.28
	(20-40)	7.34 ± 0.44	5.83 ± 0.23	5.67 ± 0.27
	P (mg kg^-1^) (0-10)	31.55 ± 0.77	23.67 ± 0.3	7.15 ± 0.55
	(10-20)	28.42 ± 2.61	17.65 ± 0.46	4.77 ± 0.42
	(20-40)	17.47 ± 0.76	13.26 ± 0.36	3.64 ± 0.43
	Soil type [51]	chernozems	castanozems	brown desert

MAT = mean annual temperature, MAP = mean annual precipitation, N = total content of soil nitrogen, and P = total content of soil phosphorus of different layers (0 - 10, 10-20, 20-40 cm).

For each type of steppe, three 14 m × 34 m plots with approximately 100 m inter-plot distance were randomly set up in order to decrease the influence of spatial heterogeneity on vegetation structure and function (Figure 1). For each plot, there were 36 subplots, 2 m × 2 m in size, with 2 m distance between any two adjacent subplots (Figure 1). We took samples in the 1 m × 1 m quadrat located at the center of each subplot. We are aware that the three plots per site were not entirely independent in comparisons between sites, but such pseudoreplication was unavoidable as the three steppe types followed a single north-south gradient. We believe that the large distances between plots within sites served to capture at least some of the possible topographic and edaphic heterogeneity that would have been expected if three replicate gradients had been compared.

### Measurements

A total of 324 quadrats were surveyed in mid-August, 2009 to examine steppe composition and structure. We measured the abundance and the cover percentage of each species in each quadrat. Species density was determined by dividing the number of individuals or tussocks by the quadrat area; a species’ cover was determined as the proportion of the quadrat area covered by its canopy. Moreover, we summarized the species frequency as the number of quadrats with at least one individual divided by the total number of the sampled quadrats in a plot. Using those three measures above, the importance value of a species was the mean value of its relative density, relative cover and relative frequency using its absolute values divided by the sum of the densities, cover proportions and frequencies of all species in a plot, respectively.

Additionally, we determined the species richness, diversity and evenness of each quadrat. Species diversity was calculated using Simpson’s reciprocal index (*D*), where $D=1/\sum_{s=1}^{s}P_{s}^{2}$and *P*_s_ is the abundance of species *s* divided by the total number of individuals from all species in a quadrat. This index can overcome the problem of the counter-intuitive nature of commonly used Simpson’s index and its value ranges from 1 to the maximum (the number of species in a quadrat). Species evenness (*E*_var_) was calculated with Camargo’s index, where $E_{\mathrm{var}}=1-2/\pi \cdot \arctan\{\sum_{s=1}^{s}(\ln(x_{s})-\sum_{s=1}^{s}\ln(x_{s})/{S)}^{2}/S\}$ and *x*_*s*_is the abundance of species s [30]. We chose *E*_*var*_ for species evenness due to its independence of the number of species in a sample [31].

We took soil samples with the soil auger of 3 cm diameter from three soil depth layers (0 - 10, 10-20 and 20-40 cm) in each quadrat. The five 1 m × 1 m quadrats in each plot were randomly chosen and the above-ground biomass was harvested at the end of August, 2009. These plant materials were oven-dried (at 65°C for 48h) and weighed in the lab. The obtained data served to calculate above-ground net primary productivity (ANPP) as peak above-ground dry mass in one year divided by the quadrat area (g m^-2^ yr^-1^). This calculation overestimates ANPP of shrubs, which have perennial (woody) structures aboveground, but since their biomass was close to negligible compared to that of the herbaceous species (Table 2) this was not considered a problem. Total contents of soil N and P were determined by Kjeldahl and H_2_SO_4_-HClO_4_ methods, respectively [32].

**Table 2:** Means of importance values (± SE) of the top five dominant species in the meadow, typical and desert steppes.

Species	Plant functional type	Importance value
**Meadow steppe**		
*Potentilla acaulis*	early-season perennial forb	18.1 ± 6.1
*Festuca lenensis*	early-season perennial grass	6.6 ± 1.2
*Carex pediformis*	early-season perennial sedge	5.8 ± 1.1
*Aster alpinus*	Late-season perennial forb	5.6 ± 1.1
*Carex duriuscula*	early-season perennial sedge	4.9 ± 0.4
**Typical steppe**		
*Salsola collina*	late-season annual forb	19.5 ± 1.2
*Stipa grandis*	late-season perennial grass	11.2 ± 0.3
*Artemisia palustris*	late-season annual forb	9.1 ± 0.7
*Elymus chinensis*	early-season perennial grass	5.7 ± 1.0
*Artemisia frigida*	late-season perennial forb	5.2 ± 0.3
**Desert steppe**		
*Allium polyrrhizum*	late-season perennial forb	19.1 ± 2.5
*Carex duriuscula*	early-season perennial sedge	13.9 ± 1.1
*Stipa gobica*	early-season perennial grass	13.2 ± 1.6
*Artemisia frigida*	late-season perennial forb	10.1 ± 0.6
*Allium anisopodium*	late-season perennial forb	7.6 ± 0.4

Plant functional type (PFT) is defined as an aggregation of species with strong functional similarity based on their role in the ecosystem and their use of resources [33]. PFT diversity was considered as a proxy for functional diversity in our study. Here PFTs were established by all combinations of growth form (shrub, grass, sedge and forb), flowering phenology (early-season versus late-season according to whether flowering occurred before July or not) and plant lifespan (annual versus perennial), only 10 PFTs of which exist in steppe ecosystems based on our surveyed species list. The importance value of a PFT was the sum of the importance values of its member species in a plot. Similarly, the abundance of a PFT was the sum of the abundances of its member species in a quadrat. These indicate the importance value of a species or a PFT was evaluated at the plot scale while diversity indices were evaluated at the quadrat scale.

### Data analysis

We calculated plant diversity indices including richness, diversity, and evenness in terms of species and PFT (based on both all species and the top five abundant species) in each of the quadrats. For each steppe type, one-way analysis of variance (ANOVA) was employed to test the differences in composition among top five dominant species or among ten PFTs, respectively, followed by Tukey’ Honestly Significant Difference tests. A similar procedure was performed to examine the overall difference in plant diversity indices or ANPP among steppe types. For each steppe type, ordinary least square regression was performed to examine whether there was a significant relationship between species richness and PFT richness or between species diversity and PFT diversity. ANCOVA was then applied to test the homogeneity among these slopes [34]. In addition, we used random resampling to determine how much variation in the relationship between species richness and PFT richness would occur by chance [35]. The resampling procedure was that species were drawn at sample sizes 2-40 (simulated species richness) from the regional species pool (113 species) and then PFT richness in the species sample was summarized. The number of iterations was 1000. The 2.5 and 97.5% quantiles of simulated PFT richness were obtained based on random samples using the 'quantile' function in R, respectively. The relationship between simulated species richness and quantiles of simulated PFT richness was fitted by power function using the 'nls' function in the 'nlme' package. For any statistical test, we chose a significance level of 0.05. Data on importance values were log_10_(x+1) transformed to meet normality and homogeneity of ANOVA [36]. All data analyses were done with R2.15.0 software [37].

## Results

### Composition and structure of the Mongolian steppe

**Figure 2:**
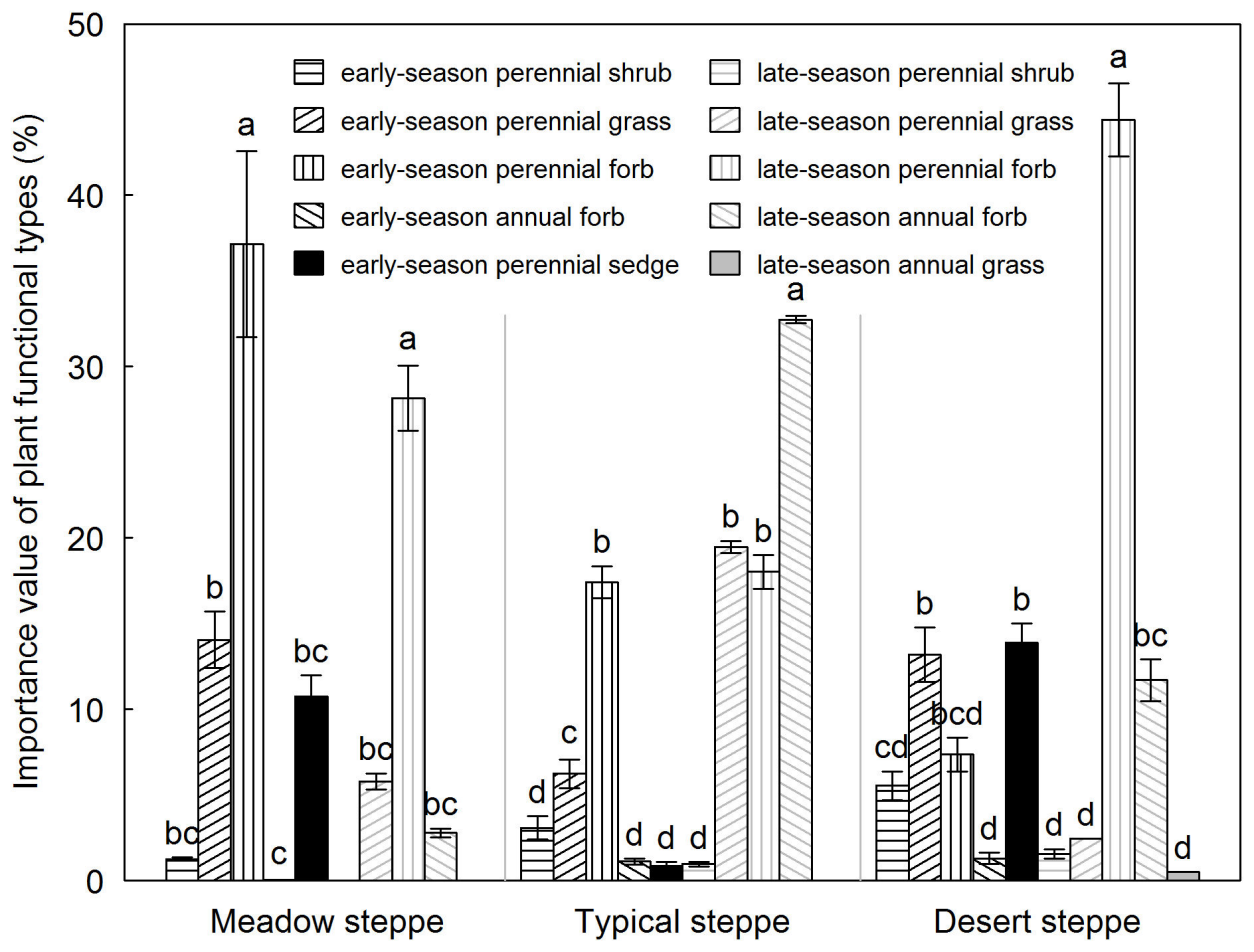
Bar diagram of importance value of ten plant functional types for each of three types of steppes (mean ± SE). Different lower case letters among ten plant functional types denote significant difference of importance value at *P* < 0.05.

**Figure 3:**
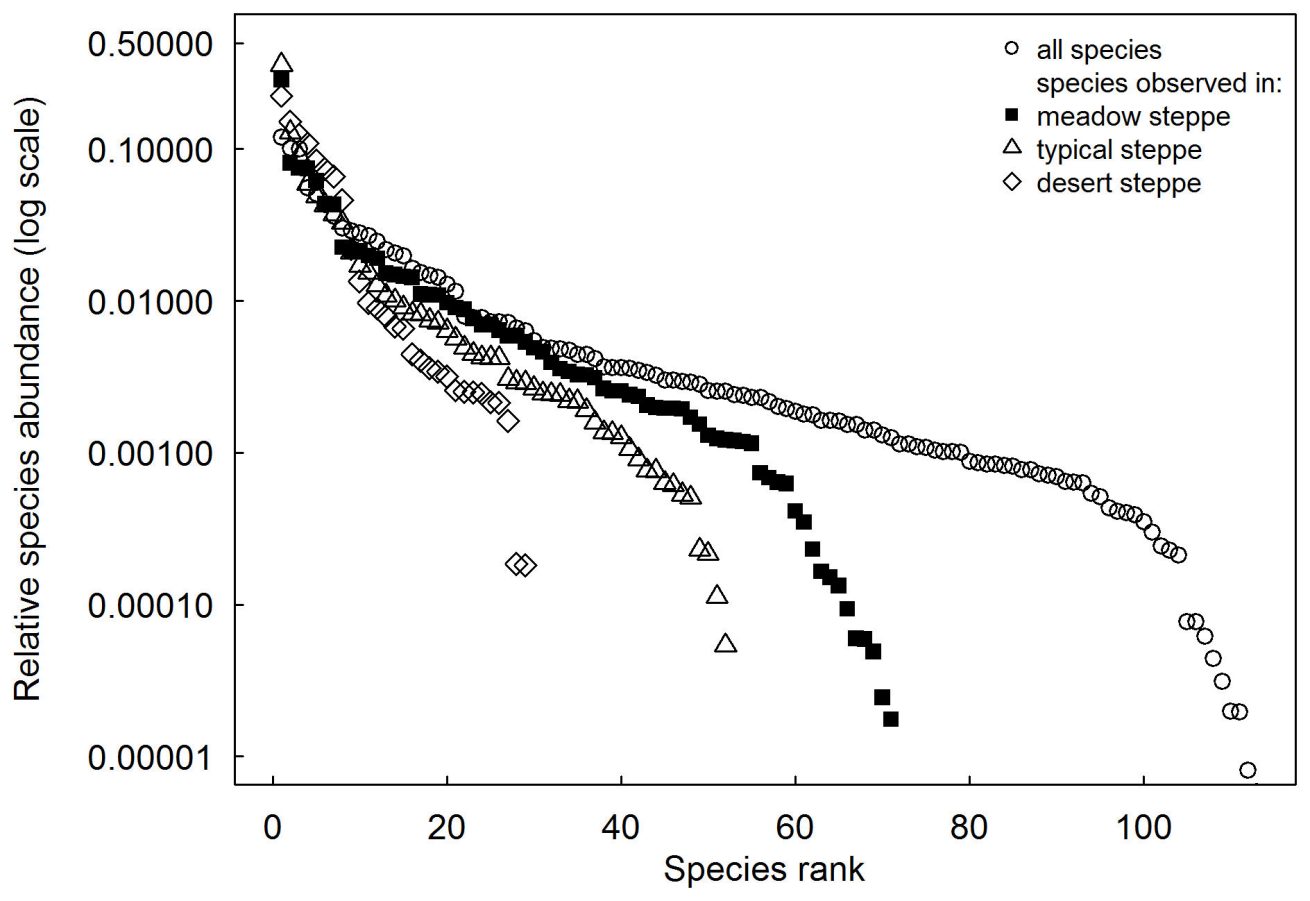
Simple rank- abundance curves for three types of steppes and for all steppe types summed.

The three types of steppes differed in species identities and PFTs of their plant communities (Table 2, Figure 2). For meadow steppe, the early-season and late-season perennial forbs explained respectively 37 and 28% of total importance value. The former consisted mainly of *Potentilla acaulis* (Table 2, Figure 2). Moreover, there was no shrub species in this steppe type (Table 2). The late-season annual forb (comprising mostly *Salsola collina* and *Artemisia palustris*) dominated in typical steppe. The dominant PFTs in meadow steppe were subdominant in typical steppe (Table 2, Figure 2). In contrast, the late-season perennial forbs (comprising mainly *Allium polyrrhizum*, *Artemisia frigida* and *Allium anisopodium*) in desert steppe accounted for 44% of the total importance value; the early-season perennial grasses, sedge and the late-season annual forbs were subdominant in this steppe type (Table 2, Figure 2). The rank-abundance curve showed that species characteristic of desert steppe had a smaller species pool with fewer minor species (i.e. a steep gradient) than meadow and typical steppes (Figure 3).

### Patterns in species, PFT diversity and evenness

Desert steppe had the lowest species richness, diversity and PFT richness among three types of steppes (Table 3). However, the desert and meadow steppes had a greater PFT diversity (in terms of all species or top five abundant species) than typical steppe (Table 3); the largest species evenness and PFT evenness always appeared in desert steppe. In contrast to evenness, desert steppe had far lower ANPP than meadow and typical steppes (Table 3).

**Table 3:** Plant diversity indices and above-ground net primary productivity (ANPP) of three steppes (mean ± SE).

Index	Meadow steppe	Typical steppe	Desert steppe
Species richness	26.90 ± 0.41a	17.10± 0.30b	9.09 ± 0.21c
Plant functional type (PFT) richness	6.11 ± 0.06a	6.26 ± 0.10a	5.32 ± 0.10b
PFT richness of top five abundant species	3.81 ± 0.06a	3.38 ± 0.06b	3.42 ± 0.07b
Species diversity	7.11 ± 0.36a	5.39 ± 0.22b	5.29 ± 0.16b
PFT diversity	3.27 ± 0.09a	2.83± 0.08b	3.17 ± 0.08a
PFT diversity of top five abundant species	3.11 ± 0.11b	3.08 ± 0.08b	3.85 ± 0.07a
Species evenness	0.41 ± 0.01c	0.45 ± 0.01b	0.58 ± 0.01a
PFT evenness	0.42 ± 0.02b	0.42 ± 0.01b	0.54 ± 0.02a
PFT evenness of top five abundant species	0.70 ± 0.02b	0.69 ± 0.01b	0.78 ± 0.02a
ANPP (g m^-2^ yr^-1^)	105.28±4.98a	121.54±9.37a	9.91 ±1.15b

Different lower case letters among three steppes denote significant difference at *P* < 0.05.

### Correlation between species diversity and PFT diversity

**Figure 4:**
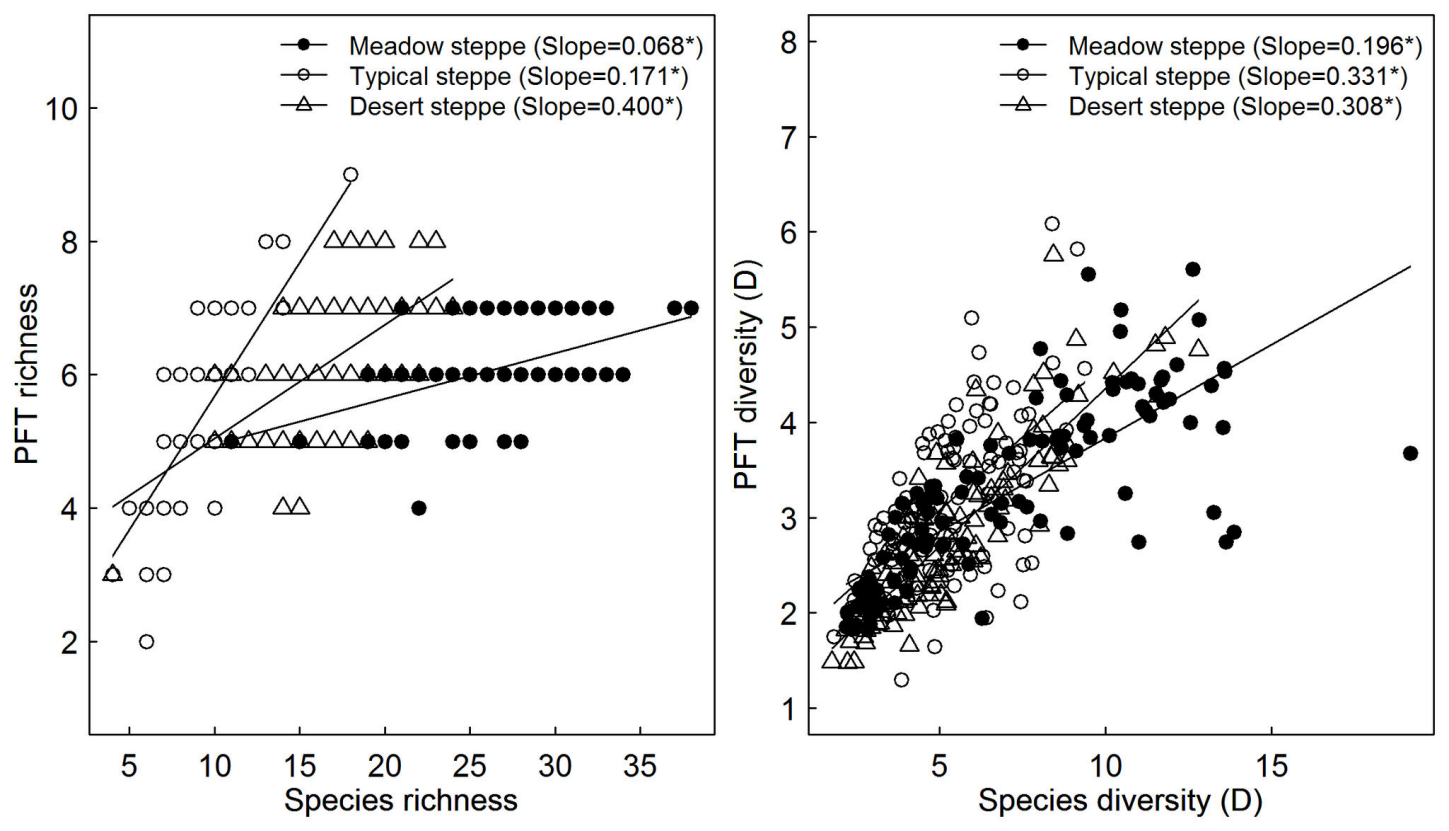
The relationships between species richness and plant functional type (PFT) richness (left panel) or between species diversity (Simpson’s reciprocal index (D), right panel) and PFT diversity for three types of steppes. *denotes a significant slope at *P* <0.05.

**Figure 5:**
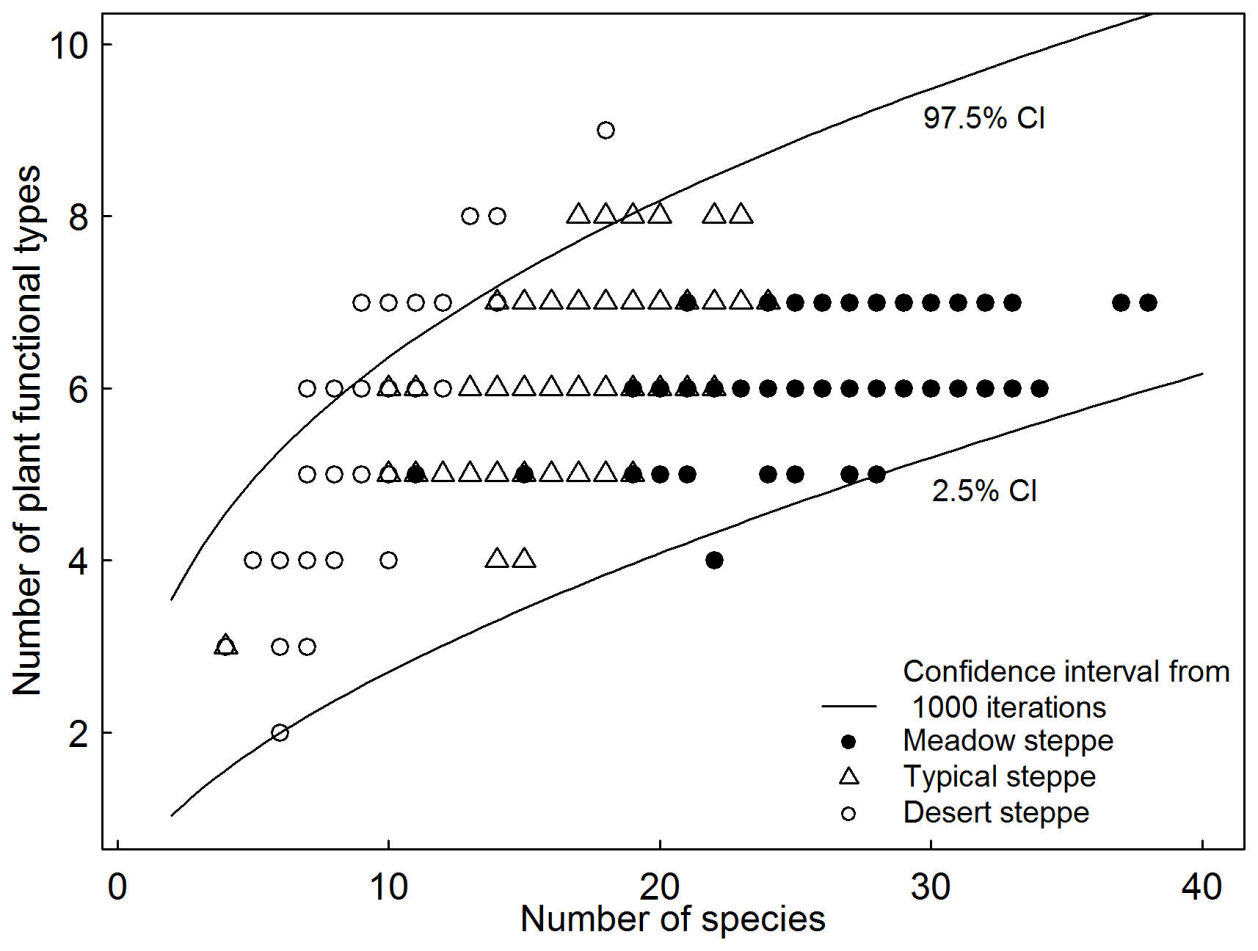
A scatter plot with points determined by species richness and plant functional type (PFT) richness in each of three types of steppes. Two solid lines were fitted by power function based on 2.5 and 97.5% quantiles derived from samples by drawing species at sample sizes 2-40 (simulated species richness) for 1000 iterations from the regional species pool, respectively.

PFT richness increased with an increase in species richness in each steppe type (Figure 4, left panel). ANCOVA indicated that the slope of relationship between species richness and PFT richness increased significantly from the meadow, via typical to desert steppe (Figure 4, left panel). Similarly, the slope of the relationship between species diversity and PFT diversity was larger in desert and typical steppes than that in meadow steppe (Figure 4 right panel). Almost all observed quadrats in the meadow and typical steppes fell within boundaries of the 95% confidence interval derived from random resampling. In contrast, approximately 30% of the surveyed quadrats in desert steppe were not within this range (Figure 5).

## Discussion

### Functional diversity sensitivity to species loss in desert steppe

This study showed that both species richness and PFT richness in desert steppe were far lower than that in meadow and typical steppes. This coincides with lower annual precipitation and low soil nutrient availability in desert steppe, which constrain species’ invasion success [38]. However, the number of functional types in desert steppe was a little larger relative to the meadow and typical steppes, indicating that quadrat-to-quadrat variation in PFT composition resulted in lower PFT richness. This could also be explained by the high soil heterogeneity in desert steppe based on both our data and previous studies [39,40]. In contrast, higher species evenness and PFT evenness in desert steppe seem to conflict with its higher spatial resource heterogeneity (see above). The likely reason for this is that desert steppe has fewer rare species than the meadow or typical steppes [41].

We found that PFT richness in desert steppe increased more quickly with an increase in species richness due to limited species redundancy. For instance, species richness in desert steppe was 9.09 ± 0.21 at the 1 m × 1 m quadrat scale but PFT richness reached up to 5.32 ± 0.10 owing to the lower species number per PFT. This indicates there is limited functional redundancy in desert steppe. The functional diversity and integrity of this steppe type can therefore decline drastically once the keystone species are lost from this sensitive steppe owing to human overexploitation or climate changes, or both, in the future [18]. Declining functional diversity in desert steppe vegetation could be reversed when overexploitation is brought to a halt, based on the dispersal of lost or new species from the regional species pool. Moreover, facilitative interspecific relationship aiding successful establishment of new species often occurs in harsh environments, such as desert steppe [42]. However, that process may still depend on a nurse or keystone species associated with species’ invasion success [43]. Hence, desert steppe vegetation should be strictly protected because of their sensitivity of functional diversity to species loss.

### Preservation and utilization strategies for meadow and typical steppes

The intermediate-disturbance hypothesis asserts that plant community diversity is maximized when ecological disturbance is at intermediate frequencies or intensities or both [44]. In general, plant species diversity may increase after moderate grazing. Although this suggests that both meadow and typical steppes could be utilized moderately, management on these two types of steppes should be different. Owing to its higher species and functional diversity, and higher spatial heterogeneity, meadow steppe can resist or buffer the detrimental effects of climate changes and/or authorgenetic activities on its structure and functioning [45,46]. Therefore, in spite of its valuable forage biomass, it should be preserved due to its high conservation value. In contrast, typical steppe has the dominant grass genus *Stipa* which is resistant to herbivory and drought [47,48] and has relatively high nutritive value of forage relative to meadow steppe [49]. These could strengthen the viewpoint that typical steppe could be utilized for grazing moderately, which is consistent with a previous hypothesis that typical steppe is more resilient to grazing than the meadow and desert steppes [50].

### Correlation between species diversity and PFT diversity

As expected, the positive correlation between species diversity and PFT diversity can be seen in each of the three types of steppes, which is consistent with the rising evidence [20-23]. However, this coordination in the meadow and typical steppes is only related with the sampling effect on diversity at the 1 m × 1 m quadrat scale. This does not indicate that the plant communities in the meadow and typical steppes are assembled at random based on only the coordination between species richness and PFT richness derived from random resampling. The driving force in processes of community assembly not only shapes species composition, but it can modulate the relative abundance of its component species. The fact that species abundance is difficult to incorporate into resampling constrains our predication about the processes underlying community assembly in steppe ecosystems. Interestingly, an unexpectedly higher proportion of sampled quadrats in desert steppe laid outside the 95% confidence intervals, further suggesting that the functional diversity in desert steppe is sensitive to species loss or species’ invasion success in its plant community.

## Conclusions

Species loss in the desert steppe is especially threatening because there is limited functional redundancy and therefore the loss of a species may result in the loss of a function critical to this sensitive ecosystem. Hence, desert steppe vegetation should be strictly protected. In contrast, although both meadow and typical steppes have high potential forage production, the management of these two types of steppes should be different: meadow steppe should be preserved due to its higher conservation value with higher species diversity and higher spatial heterogeneity, while typical steppe could be utilized moderately due to its dominant grass genus *Stipa*, which is resistant to herbivory and drought. These findings aid our utilization and preservation of steppes, particularly of those in Mongolia.
